# High Intraspecific Genetic Diversity of* Nocardia brasiliensis*, a Pathogen Responsible for Cutaneous Nocardiosis Found in France: Phylogenetic Relationships by Using* sod* and* hsp65* Genes

**DOI:** 10.1155/2018/7314054

**Published:** 2018-05-20

**Authors:** D. Kosova-Maali, E. Bergeron, Y. Maali, T. Durand, J. Gonzalez, D. Mouniée, H. Sandoval Trujillo, P. Boiron, M.-C. Salinas-Carmona, V. Rodriguez-Nava

**Affiliations:** ^1^Research Group on “Bacterial Opportunistic Pathogens and Environment”, UMR Ecologie Microbienne, CNRS 5557, INRA 1418, UCBL, Université de Lyon, VetAgro Sup, Faculté de Pharmacie, 8 avenue Rockefeller, Lyon, France; ^2^Centre International de Recherche en Infectiologie, INSERM U1111, CNRS UMR5308, ENS de Lyon, Team “Pathogenesis of Staphylococcal Infections”, Université de Lyon 1, Lyon, France; ^3^Laboratoire de Bactériologie, Institut des Agents Infectieux, Centre de Biologie et Pathologie Nord, 103 grande rue de la Croix-Rousse, 69004 Lyon, France; ^4^Departamento de Sistemas Biológicos, Universidad Autónoma Metropolitana-Xochimilco, Calzada del Hueso 1100, 04960 Ciudad de México, Mexico; ^5^Facultad de Medicina, Universidad Autonoma de Nuevo Leon, Monterrey, NL, Mexico

## Abstract

This study aims at genetic characterization and phylogenetic relationships of* Nocardia brasiliensis* focusing by using housekeeping* rrs*,* hsp65, *and* sodA* genes.* N. brasiliensis* is the species responsible for 80% of cases of actinomycetoma, one form of cutaneous nocardiosis which occurs mainly in tropical regions reaching immunocompetent patients in which the disease can lead to amputation. We analyze 36 indigenous cases of* N. brasiliensis* that happened in France. Phylogenetic analysis targeting* rrs* gene showed no robustness at phylogenetic nodes level. However, the use of a concatenation of* hsp65* and* sodA* genes showed that the tested strains surprisingly ranked in 3 well-defined genotypes. Genotypes 2 and 3 were phylogenetically closer to each other and both diverged from genotype 1 sustained by a high bootstrap of 81%. This last genotype hosts all the cases of pulmonary forms (3), the sole cerebral form, and almost all the cases of immunocompromised patients (3 out of 4). Moreover, excepting one of them, all the strains belonging to this group present a susceptibility to imipenem which is not the case in the other genotypes that rarely count among them strains being susceptible to this drug. The haplotype diversity (Hd) of* hsp65* (0.927) and* sodA* (0.885) genes was higher than that of* rrs* (0.824). For this gene, we obtained 16 polymorphic sites whereas, for* hsp65* and* sodA* genes, up to 27 and 29 were identified, respectively. This study reveals that these two genes have an important genetic discriminatory power for the evaluation of the intraspecies genetic variability of* N. brasiliensis *and they may be useful for identification purposes at species level. This study also reveals the possible existence of a new species harbored by genotype 1.

## 1. Introduction

*Nocardia* is a genus belonging to the aerobic actinomycetes group of bacteria which are Gram-positive bacilli and showing branching filamentous forms [1]. They are saprophytic ubiquitous bacteria which can be found in several environments such as fresh water and saltwater, soil, dust, decaying vegetation, and decaying fecal deposits from animals [1]. Nevertheless, these environmental bacteria can be opportunistic pathogens and lead to human infectious diseases called “nocardiosis” [2]. Nocardiosis can be discriminated into two groups: invasive infection, mainly caused by* N. asteroides, *presenting commonly as pneumonia in patients who are immunocompromised, have underlying chronic lung disease, and are with a possible dissemination to other organs [3], and cutaneous infection via a cut or abraded skin, which can be manifest clinically as (i) abscess and cellulitis, (ii) lymphangitis, (iii) skin infection secondary to dissemination, and (iv) actinomycetoma. This latter group is the most amazing infection due to their severity characterized by the presence of tumefaction, subcutaneous nodules, destructive granulomata, fistulas, and pus [2, 4].

*N. brasiliensis* is the species isolated from the majority (approximately 80%) of cases of cutaneous nocardiosis, especially in actinomycetoma [2]. This species is more commonly isolated in areas with tropical or subtropical climates such as South America, Asia, and Africa. Due to false diagnosis, rural lifestyles, and poor access to care in these countries,* N. brasiliensis* nocardiosis constitutes a real public health problem that can lead, in the absence of treatment, to amputations and death in young populations. On the basis of epidemiological surveys conducted in France, the number of cases of nocardiosis between 2000 and 2007 according to the French Nocardiosis Observatory (OFN) was 607 with* N. farcinica* and* N. nova* being the most frequent species [5]. However, no data currently exists on the phylogenetic relationships between the indigenous* N. brasiliensis* strains of tropical origin and native strains isolated in France. Routine genus/species identification of* Nocardia *was based on macroscopic, microscopic, and biochemical characteristics. The methods described by Boiron et al. [6] were used to determine the decomposition of adenine, casein, hypoxanthine, tyrosine, and xanthine. In addition to the phenotype-based methods, species-level identification is mainly genetically based, nowadays. Classically, 16S rRNA* (rrs)* gene sequencing is generally used for the species-level identification [7, 8], but it fails to discriminate among some species of* Nocardia* because it does not have enough polymorphism to differentiate them at the species level. Multilocus sequence analysis (MLSA) using concatenated sequences of several housekeeping genes such as superoxide dismutase A* (sodA)* and heat shock protein 65* (hsp65)* has been increasingly used to provide higher accuracy and discriminatory power in the molecular identification of* Nocardia* spp. [9, 10]. Indeed, a recent study seeking to identify new molecular targets shows that the polymorphism observed in the* sodA* gene sequence contains variable regions that allow the discrimination of closely related* Nocardia* species [9].

The aim of the present study was to perform a genetic characterization and assess the phylogenetic relationships of* Nocardia brasiliensis* focusing on using housekeeping* rrs*,* hsp65,* and* sodA* genes, for 36 autochthonous* N. brasiliensis *strains isolated in France and analyzed by the OFN between 2002 and 2012. Phenotypic characterization was also conducted by assessing antimicrobial resistance profiles, metabolic profiles, and culture condition.

## 2. Materials and Methods

### 2.1. Bacterial Strains and Culture Media

A collection of 36 human clinical strains of* N. brasiliensis* was studied (Table 1). All strains were identified as such, at species level by the French Nocardiosis Observatory (OFN) by genetic approach. Moreover, six* Nocardia* reference strains belonging to* N. brasiliensis* clade [9] were also used:* N. brasiliensis* ATCC 19296^T^ (unknown),* N. altamirensis* DSM 44997^T^ (karstic cave),* N. boironii* DSM 101696^T^ (pus sample),* N. iowensis* DSM 45197^T^ (garden soil),* N. tenerifensis* DSM 44704^T^ (rhizosphere), and* N. vulneris* DSM 45737^T^ (human leg wound). Prior to the assays, strains were cultured 72 hours in Bennett medium (made in the laboratory) aerobically at 37°C.

### 2.2. Growth Test on Culture Media

From 0.5 McF bacterial suspension, bacterial growth was evaluated on three culture media: (i) bromocresol purple (BCP) (Biomérieux, Marcy l'étoile), (ii) Bennett (made in the laboratory), and (iii) Middlebrook (Biomérieux, Marcy l'étoile). One hundred microliters from bacterial suspension standardized was inoculated on the different plate of culture media. The plates were incubated at 37°C and the observations were performed at 48, 72, and 96 hours.

### 2.3. Antimicrobial Susceptibility

The susceptibility of the isolates to different antimicrobials was determined by disk diffusion method with a panel of 31 antibiotics (Biorad, Marnes-la-Coquette France) on Muller Hinton E medium (Biomérieux, Marcy l'étoile, France). Susceptibility testing was done with amikacin 30 *μ*g, gentamycin 15 *μ*g, tobramycin 10 *μ*g, ciprofloxacin 5 *μ*g, levofloxacin 5 *μ*g, moxifloxacin 5 *μ*g, minocycline 30 *μ*g, doxycycline 30 *μ*g, tigecycline 15 *μ*g, cefotaxime 30 *μ*g, ceftriaxone 30 *μ*g, cefepime 30 *μ*g, cefuroxime 30 *μ*g, amoxicillin 25 *μ*g, amoxicillin + clavulanic acid 20/10 *μ*g, ampicillin 10 *μ*g, ertapenem 10 *μ*g, meropenem 10 *μ*g, imipenem 10 *μ*g, vancomycin 30 *μ*g, pristinamycin 15 *μ*g, erythromycin 15 *μ*g, trimethoprim + sulfamethoxazole 1.25/23.75 *μ*g, rifampicin 30 *μ*g, and linezolid 30 *μ*g.

From visible colonies, bacterial suspension was done in sterile water, using a cotton swab to obtain a concentration of 0.5 McFarland according to the Clinical and Laboratory Standards Institute standard M24-A2 [11]. Seeding was done according to the swab method. In this latter, the bacterial inoculum was spread on the agar using a sterile cotton swab in three different directions. The disks were dispensed with a dispenser and the plates were incubated at 37°C for 72 hours and read manually according to the thresholds defined in the recommendations of the SFM 2013 [12].

### 2.4. Substrate Degradation

The methods of Boiron et al. [6], Goodfellow et al. [13, 14], and Goodfellow and Lechevalier [15] were used to determine the decomposition of adenine, casein, and uric acid [9]. Clinical strains of* N. brasiliensis* and the strains of species belonging to the* N. brasiliensis* clade (*N. brasiliensis, N. altamirensis, N. iowensis, N. tenerifensis*,* N. boironii,* and* N. vulneris*) were tested [9]. Strains* N. boironii* DSM 101696^T^,* N. brasiliensis* ATCC 19296^T^, and* N. vulneris* DSM 45737^T^ were incubated at 37°C, and* N. altamirensis* DSM 44997^T^,* N. tenerifensis* DSM 44704^T^, and* N. iowensis* DSM 45197^T^ were incubated at 28°C [9]. The readings were performed at 3, 7, 10, 14, 17, and 21 days.

### 2.5. Methods of DNA Extraction

DNA extraction from* Nocardia* strains was performed with achromopeptidase according to the method reported by Rodríguez-Nava et al. [10]. Colonies were picked off with a loop, and one loopful was suspended in 200 *μ*L of sterile water containing a dozen glass beads and vortexed for 5 minutes. The mixture was then incubated for 15 minutes at 70°C. The suspension supplemented with 3.4 *μ*L of achromopeptidase (Sigma, Steinheim, Germany) at 10 U/mL was incubated at 55°C for 15 minutes. The suspensions were then centrifuged for 5 minutes at 13,000 rpm. The supernatants were stored at −20°C until use.

### 2.6. Amplification and Sequencing

*Gene rrs.* A 606-bp fragment of the rrs gene was amplified with primers Noc1, 5′-GCTTAACACATGCAAGTCG-3′, and Noc2, 5′-GAATTCCAGTCTCCCCTG-3′, and PCR program and reaction mixture were carried out according the recommendations of Rodríguez-Nava et al. [10].

*Gene hsp65.* A 441-bp fragment of the* hsp65* gene encoding the 65-kDa heat shock protein was amplified with primers described by Telenti et al. (TB11: 5′-ACCAACGATGGTGTGTCCAT-3′ and TB12: 5′-CTTGTCGAACCGCATACCCT-3′) [16]. PCR program and reaction mixture were carried out according to the recommendations of Sánchez-Herrera et al. [17]. 

*Gene sodA.* A 440-bp fragment of the* sodA* gene was amplified and sequenced with primers SodV1 (5′-CAC CAY WSC AAG CAC CA-3′) and SodV2 (5′-CCT TAG CGT TCT GGT ACT G-3′) where Y = C or T, W = A or T, and S = C or G. The amplification was also done according to the recommendations of Sánchez-Herrera et al. [17].

All resulting PCR products were sequenced and verified (Biofidal, Lyon, France).

The breakpoints for identification based in* sodA* and* hsp65* genes are 99% for each one [17, 18]. For the* rrs* gene, a higher breakpoint of 99.6% is used, according to CLSI [19].

### 2.7. Phylogenetic Analysis

The* rrs* gene sequences which we obtained for the 36 clinical isolates of* N. brasiliensis* and the reference strains were aligned manually for the comparative phylogenetic analysis using the Seaview program.

MLSA was performed using* hsp65* and* sodA* sequences of the strains collection. The trimmed aligned sequences were concatenated in the order* sodA-hsp65* to generate an 846 bp sequence using the Seaview program. The Seaview program was also used to infer the evolutionary trees according to the neighbour-joining method [20] and Kimura's two-parameter model [21]. The robustness of the tree was performed with a bootstrap of 1000 replicates.

Taking into account the breakpoints for identification at species level of* sodA* and* hsp65* genes individually, the breakpoint for concatenated sequence has been also fixed at 99%.

### 2.8. DNA Polymorphism of* rrs*,* hsp65,* and* sod*A Genes

The number of haplotypes, the haplotype diversity (Hd), the number of polymorphic sites, and other variables were obtained with DnaSP software [22].

## 3. Results

### 3.1. Growth on Culture Medium

The three culture media allowed the growth of clinical strains of* N. brasiliensis*. The Bennett medium showed abundant and rapid growth (48 hours). Middlebrook medium showed strong growth but also it was slightly slower (72 hours). The BCP medium presented interesting results with good rapid growth at 48 hours.* N. brasiliensis* clade tested type strains showed similar patterns to the clinical strains, except that* N. boironii* had a difficult growth on BCP and no growth on Middlebrook; this seems a peculiarity of this species.

### 3.2. Antimicrobial Susceptibilities

Eight out of 31 antibiotic molecules tested were active on all the strains' collection: linezolid, tigecycline, trimethoprim + sulfamethoxazole, moxifloxacin, amikacin, amoxicillin + clavulanic acid, tobramycin, and gentamycin. Regarding the imipenem and pristinamycin molecules, resistance was observed on the majority of clinical isolates of* N. brasiliensis *(Table 1).

### 3.3. Degradation of Substrate

The assimilation test of adenine and uric acid proved negative for all the strains tested of the* N. brasiliensis* clade including clinical and reference ones. The casein degradation test showed that all clinical strains are able to metabolize casein except the clinical strain 12.28. In addition our result showed that some types of strains such as* N. vulneris*,* N. tenerifensis*,* N. boironii,* and* N. iowensis* are also able to degrade casein in the same way as* N. brasiliensis* except* N. altamirensis*. Casein is ultimately a marker that can be used for the phenotypic identification of the* N. brasiliensis* clade and not the* N. brasiliensis* species as it has been believed for many years.

### 3.4. Phylogeny

**Figure 1:**
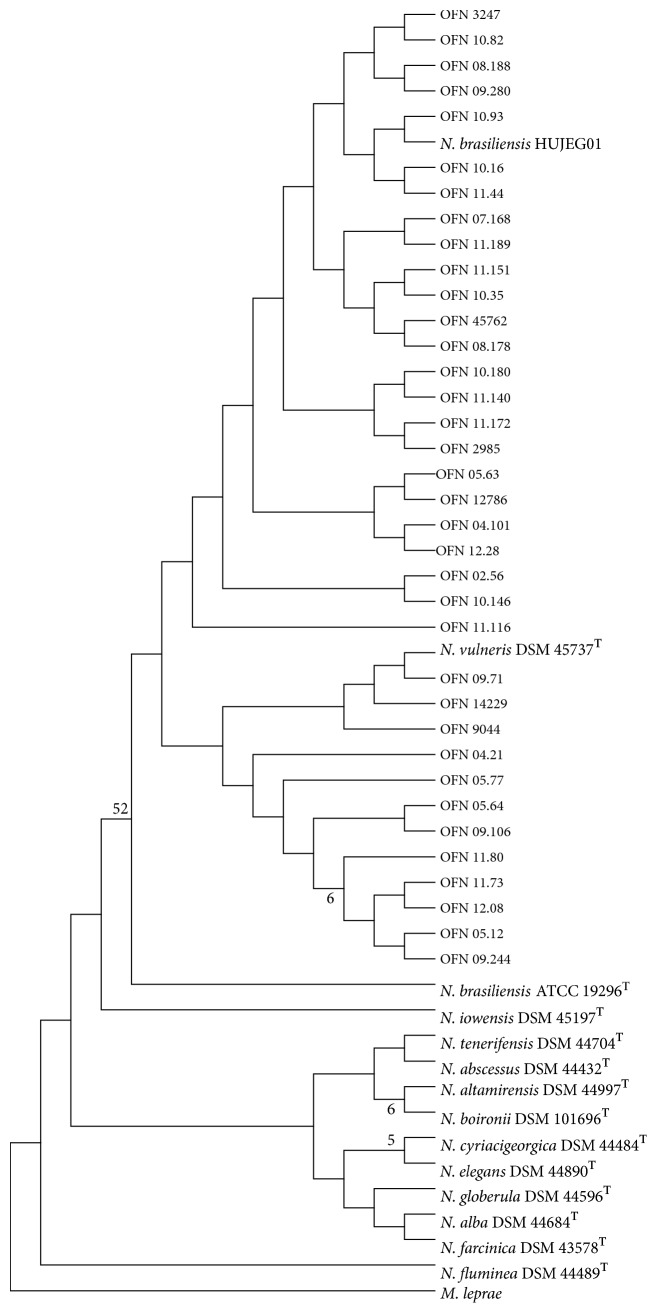
Phylogenetic distribution of* rrs* gene of 36* N. brasiliensis *clinical strains analyzed in this study using neighbour-joining method, Kimura's two-parameter model, and bootstrap of 1000. Only values of bootstrap significance greater than 50% (Seaview) were reported.

Primers Noc1 and Noc2 amplified the expected 606-bp fragment of the* rrs* gene for all the collection strains. Phylogenetic trees (Figure 1) based upon* rrs* showed homogeneity within clinical strains of* N. brasiliensis*. For this, the* rrs* gene is not relevant to show intraspecies diversity.

**Figure 2:**
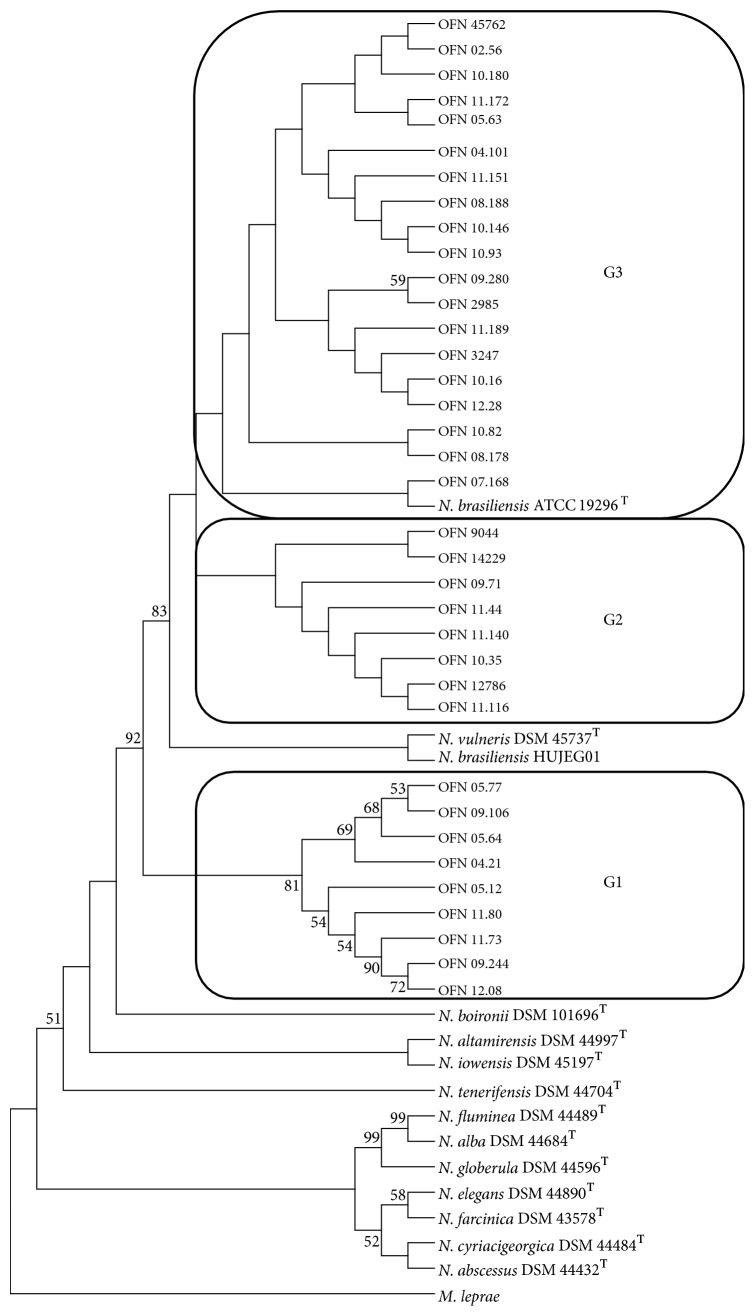
Phylogenetic distribution of concatenation* sodA-hsp65* genes of 36* N. brasiliensis *clinical strains analyzed in this study using neighbour-joining method, Kimura's two-parameter model, and bootstrap of 1000. Only values of bootstrap significance greater than 50% were reported.

In addition, based upon the concatenation of* sodA* and* hsp65* housekeeping genes, the phylogenetic tree generated (Figure 2) had several distinct genotypes: (i) genotype 1 containing clinical strains, (ii) genotype 2 harboring some clinical strains, and (iii) genotype 3 harboring some clinical strains and* N. brasiliensis* ATCC 19296^T^. For the tropical* N. brasiliensis *HUJEG01 strain, it is observed that it does not belong to any of the 3 genotypes and is positioned alone in the tree between genotypes 1 and 2. This distribution of clinical strains of* N. brasiliensis* in 3 different genotypes shows an intraspecies diversity rather important. To better understand the polymorphism showed by phylogenetic trees, we studied the percentages of the similarities between the sequences. The average percentages of similarities based on the* rrs* gene (Table 2) range from 99.39% to 99.57% between the clinical strains and the 2 reference strains of* N. brasiliensis* (type and tropical strains). According to the CLSI, the similarity percentage needed for identification at species level must be greater than or equal to a threshold of 99.6% [19]. The clinical strains that showed a similarity percentage lower than this threshold for both reference strains of* N. brasiliensis* were anyway considered as belonging to this species because no higher similarity percentage was obtained for any other species. In the same way, the* N. vulneris* type strain was also revealed to be close to clinical strains according to the average of percentage of similarity (98.77%). Between the 2 reference strains of* N. brasiliensis* the percentage of similarity is higher, up to 99.82%. The percentages of similarities based on the concatenation of the* sodA-hsp65* genes (Table 2) decrease and range now from 97.99% to 99.19% between the clinical strains and the 2 reference strains of* N. brasiliensis*. Between the type and reference strains of* N. brasiliensis* the percentage of similarity does not reach 99% this time. The comparison of the 3 genotypes between them (based on the representation of each genotype by 3 clinical strains) by using* sodA-hsp65* genes shows that genotypes 2 and 3 are closer to each other (98.97% of similarity). The average of the percentages of similarity between genotypes 1 and 2 were 97.97%. and 98.28% between genotypes 1 and 3. Finally this value goes up to 98.97% between genotypes 2 and 3. This means that the more distant genotypes between them are 1 and 2 and the closer ones are 2 and 3.

In parallel, an epidemiological study based on the clinical files was carried out, and the data were presented in Table 1. In order to know the link between the genetic diversity and the tropism of the clinical strains, a superposition of data was made between the phylogenetic tree obtained by the concatenation of* sodA* and* hsp65* and the tropism of the clinical strains (Figure 2). Thus, we can see that in genotypes 2 and 3 we have almost all the clinical strains that have a cutaneous tropism except the 08.188 strain which has a subcutaneous tropism. Regarding genotype 1 it is more heterogeneous with various tropism: (i) pulmonary, (ii) cerebral, and (iii) cutaneous. Regarding the immunocompetence of patients, we have only 4 patients who have immunodepression factors, whose strains are in genotype 1 except the 10.93 strain which is in genotype 3.

### 3.5. Analysis of* rrs*,* hsp65,* and* sod*A Genes Polymorphism

The 36 clinical strains and 2 reference strains of* N. brasiliensis* studied showed (i) for* rrs* gene 16 polymorphic sites sharing 16 haplotypes and showing a Hd of 0.824; (ii) for* hsp65* gene, 27 polymorphic sites and up to 22 different haplotypes with a Hd of 0.927; and, (iii) for* sodA* gene, up to 29 polymorphic sites sharing 14 haplotypes having a Hd of 0.885 (Table 3).

## 4. Discussion

*Nocardia* spp. are common soil-inhabiting bacteria that frequently infect humans through traumatic injuries or inhalation routes and cause infections, such as actinomycetoma and nocardiosis, respectively.* N. brasiliensis* is the main aetiological agent of actinomycetoma in various countries [23]. The input data used in this study highlight the existence of indigenous cases of cutaneous and subcutaneous (such as actinomycetoma) nocardiosis caused by* N. brasiliensis* in France. Moreover, we can observe that* N. brasiliensis* is also responsible for severe cases of disseminated nocardiosis in immunocompromised patients (pulmonary and cerebral cases).

To determine whether there is an association between clinical tropism of strains and their genetic profile we performed genetic characterization of 36 indigenous cases of* N. brasiliensis* that happened in France.

The three culture media allow the growth of clinical strains of* N. brasiliensis*. However, on Bennett's medium more abundant and fast growth (48 hours) was observed. But the downside of this medium is its inaccessibility in the hospital because it is not marketable. Middlebrook medium shows strong growth but also it was slightly slower (72 hours). This medium is very expensive and not accessible to all budgets. However, it is an interesting alternative in isolating* Nocardia* from a complex sample. It is a selective medium of Mycobacteria, which promotes the growth of some* Nocardia* to the detriment of other external bacteria or commensal flora that may be in the biological sample analyzed. The BCP medium, used routinely in hospitals for Gram-negative bacteria, has interesting results with good fast growth (48 hours). It would therefore be advisable to use it as isolation medium for urgent cases, by the speed of growth.

Antibiograms results show resistance of most of the clinical strains to imipenem. This can pose therapeutic problems since it is part of molecules proposed during a phase of a general treatment for nocardiosis [24]. However, all clinical strains of* N. brasiliensis* were sensible to SXT and would be an effective molecule during treatments. The sensibility of* N. brasiliensis* type strain to this antibiotic has already been observed by Gilquin et al. [9].

Our study confirms that all clinical strains of* N. brasiliensis* are capable of degrading casein except 12.28 clinical strain. As shown by Seol et al. the* N. brasiliensis* type strain is able to degrade casein as well [25]. However, the test on the reference strains reveals that* N. vulneris*,* N. tenerifensis*,* N. boironii,* and* N. iowensis* are also capable of degrading casein in the same way as* N. brasiliensis,* as also shown by Gilquin et al. [9]. This type of test is used in some countries without the necessary molecular biology tools to identify* N. brasiliensis*. But, now, they must be aware that with this test we target several species of clade* N. brasiliensis*. So, it is no longer a criterion of identification proper to* N. brasiliensis*.

Phylogenetic tree based on the* rrs* gene sequence of our collection showed a low genetic diversity resulting in low polymorphism sequence. In addition, we can note that* N. vulneris* DSM 45737^T^, identified as a new species by Lasker et al., present a genetic sequence very close to* N. brasiliensis* strains [26] with percentages of similarities on average greater than 98%.

Analysis of the phylogenetic tree (Figure 2) based on the MLSA by the concatenation of* sodA* and* hsp65* housekeeping genes showed that the isolates are surprisingly classified according to 3 genotypes. These groups were formed upon similarity percentages and existing phylogenetic distances between the sequences of the strains studied. Genotype 1 concerns a well-defined cluster containing 9 clinical strains only which is sustained by a bootstrap of 81%. This genotype hosts all the cases of pulmonary forms (3), the sole cerebral form, and almost all the cases of immunocompromised patients (3 out of 4). Moreover, eight out of twelve strains susceptible to imipenem can be found in this group. There is just one remaining strain in this group not presenting this kind of susceptibility. The reason may be an acquired resistance to this drug due to a previous treatment. This well-defined genotype evokes the possible existence of another species or a strong variability in this case. This may have been caused by environmental pressures in the ecosystem of these isolates which may have resulted in the selection of strains that may have acquired, by mutations or genetic transfer with other microorganisms, new virulence characters different from that of the strain type* N. brasiliensis*. Regarding genotypes 2 and 3, they include clinical strains and* N. brasiliensis* ATCC 19296^T^ type strain. However, the discrimination between these 2 genotypes is less clear than that with genotype 1 because of being in weak bootstrap that is less than 50. The genetic differences do not allow distinguishing them properly and their phenotypic behavior remains similar. Then, it would be interesting to study on another gene capable of generating more divergences, for example,* gyrB* and* rpoB* genes, which have already successfully been used for studying the polymorphism of some other* Nocardia* species [27, 28].

Concerning the percentage of similarity between the type and tropical strain of* N. brasiliensis*, it is 99.82% according to the* rrs* and goes down to 98.51% with the concatenation of* sodA* and* hsp65*. The fact of highlighting a greater dissimilarity with the concatenation between the type and tropical* N. brasiliensis* strain shows the advantage of the use of 2 markers like* sodA* and* hsp65* vis-à-vis the* rrs*. The discriminatory power of these two genes may be explained by the presence of more polymorphic sites (*hsp65*: 27;* sodA*: 29) than in the case of* rrs* gene (16) and also by having Hd values higher than that of* rrs* gene (*hsp65*: 0.927;* sodA*: 0.885;* rrs*: 0.824).

It would be interesting to identify the genes involved in the virulence of different genotypes, including those of actinomycetoma. Interesting leads can be considered: (i) as identification of virulence genes expressed using the RNAseq method or (ii) to identify noncoding RNAs [23]. In addition, to genomically distinguish* N. brasiliensis* and* N. vulneris* a specific PCR to* N. vulneris*, using a specific gene of the species, should be developed.

## Figures and Tables

**Table 1:** Table of clinical strains including the type of tropism observed in host, their corresponding *sodA/hsp65* genotypes, and drug phenotypes.

Sample date	Nature of sampling	Patient record	Immunosuppressed	Genotype *sodA/hsp65*	Tropism	Pristinamycin	Imipenem	Amikacin	Trimethoprim + sulfamethoxazole
04/2002	Intraoperative tissue	02.56	No	G3	Cutaneous	R	R	S	S
12/2003	Pus from cutaneous thigh abscess	04.21	Yes	G1	Cutaneous	R	S	S	S
2004	Pus from cutaneous abscess	04.101	No	G3	Cutaneous	R	R	S	S
07/2005	Expectoration then LBA	05.64	Yes	G1	Lung	R	S	S	S
01/2005	Skin biopsy	05.12	No	G1	Cutaneous	R	S	S	S
07/2005	Phalanx biopsy	05.63	No	G3	Cutaneous	R	R	S	S
2005	CSF	05.77	No	G1	Brain	R	R	S	S
11/2005	-	3247	No	G3	Unknown	R	S	S	S
2007	Cutaneous abscess	07.168	No	G3	Cutaneous	R	R	S	S
10/2008	Wound of forehead	08.178	No	G3	Cutaneous	R	S	S	S
11/2008	Subcutaneous abscess	08.188	No	G3	Subcutaneous	R	R	S	S
10/2008	Elbow abscess	2985	No	G3	Cutaneous	R	R	S	S
03/2008	-	9044	No	G2	Unknown	R	R	S	S
03/2009	Pus of leg abscess	09.71	No	G2	Cutaneous	R	S	S	S
04/2009	Bronchial aspiration	09.106	No	G1	Lung	R	S	S	S
10/2009	Bronchial aspiration	09.244	Yes	G1	Lung	R	S	S	S
12/2009	Finger skin	09.280	No	G3	Cutaneous	R	R	S	S
10/2009	Pus from the lip	10.16	No	G3	Cutaneous	R	R	S	S
02/2010	Finger abscess	10.35	No	G2	Cutaneous	R	R	S	S
05/2010	Hand abscess	10.82	No	G3	Cutaneous	R	R	S	S
05/2010	Toe abscess	10.93	Yes	G3	Cutaneous	R	R	S	S
09/2010	Leg wound	10.146	No	G3	Cutaneous	R	R	S	S
11/2010	Pus finger	10.180	No	G3	Cutaneous	R	R	S	S
11/2010	Leg wound	12786	No	G2	Cutaneous	R	R	S	S
04/2010	-	14229	No	G2	Unknown	R	S	S	S
07/2010	Sepsis hand	45762	No	G3	Cutaneous	R	R	S	S
2011	Pus	11.44	No	G2	Cutaneous	R	R	S	S
05/2011	Cutaneous abscess	11.73	No	G1	Cutaneous	R	S	S	S
05/2011	Hand wound	11.80	No	G1	Cutaneous	R	S	S	S
08/2011	Cutaneous	11.116	No	G2	Cutaneous	R	R	S	S
2011	Thigh abscess	11.140	No	G2	Cutaneous	R	R	S	S
09/2011	Knee wound	11.151	No	G3	Cutaneous	R	R	S	S
2011	Hand wound	11.172	No	G3	Cutaneous	R	R	S	S
12/2011	Cutaneous abscess	11.189	No	G3	Cutaneous	R	R	S	S
2012	-	12.08	No	G1	Unknown	R	S	S	S
02/2012	Cutaneous abscess	12.28	No	G3	Cutaneous	R	R	S	S

**Table 2:** Percentage of similarity expressed in interval and mean for *sodA-hsp65* and *rrs* genes.

		*N. brasiliensis* HUJEG01		*N. brasiliensis* ATCC 19296${}^{\overset{\;}{T}}$		*N. vulneris *DSM 45737${}^{\overset{\;}{T}}$	
		*sodA-hsp65*	*rrs*	*sodA-hsp65*	*rrs*	*sodA-hsp65*	*rrs*
*N. brasiliensis *ATCC 19296${}^{\overset{\;}{T}}$	Similarity average (%)	98.51	99.82	-	-	-	-
*N. vulneris *DSM 45737^T^		98.38	98.73	98.51	98.55	-	-
*N. altamirensis *DSM 44997^T^		95.91	98.24	95.41	98.07	95.91	97.47
*N. boironii *DSM 101696^T^		96.53	98.07	96.28	97.89	96.15	97.29
*N. iowensis* DSM 45197^T^		94.91	97.89	95.29	97.71	94.54	97.64
*N. tenerifensis* DSM 44704^T^		96.28	96.67	96.03	96.49	95.91	96.03

Genotype 1	Similarity range (%)	(97.77–98.38)	-	(97.77–98.26)	-	(97.52–98.14)	-
	Similarity average (%)	98.01	-	97.99	-	97.67	-

Genotype 2	Similarity range (%)	(98.39–98.88)	-	(98.88–99.38)	-	(98.63–98.88)	-
	Similarity average (%)	98.73	-	99.19	-	98.80	-

Genotype 3	Similarity range (%)	(98.14–98.88)	-	(98.26–99.00)	-	(98.01–98.76)	-
	Similarity average (%)	98.63	-	98.76	-	98.50	-

All clinical strains	Similarity range (%)	-	(98.57–100)	-	(98.38–99.82)	-	(98.01–99.09)
	Similarity average (%)	-	99.57	-	99.39	-	98.77

**Table 3:** DNA polymorphism of *rrs*, *hsp65,* and *sodA* genes from clinical *N. brasiliensis* strains isolated in France.

*Nocardia* species	Genes (bp)^a^	Number of haplotypes (Hd, S^2^, SD)^b^	Number of of polymorphic sites
*N. brasiliensis* (*N* = 38: 36 clinical strains and 2 reference strains)	*rrs* (569)	16 (0.824, 0.00300, 0.055)	16
	*hsp65 *(401)	22 (0.927, 0.00087, 0.029)	27
	*sodA* (406)	14 (0.885, 0.00080, 0.028)	29

^a^Resulting fragment size without the primers sequences; ^b^Hd: haplotype (gene) diversity, S^2^: variance of haplotype diversity, and SD: standard deviation of haplotype diversity.
